# Fuzzy cognitive mapping and soft models of indigenous knowledge on maternal health in Guerrero, Mexico

**DOI:** 10.1186/s12874-020-00998-w

**Published:** 2020-05-19

**Authors:** Ivan Sarmiento, Sergio Paredes-Solís, David Loutfi, Anna Dion, Anne Cockcroft, Neil Andersson

**Affiliations:** 1 CIET-Participatory Research at McGill, Faculty of Medicine, Department of Family Medicine, McGill University, 5858 Chemin de la Côte des Neiges 3rd floor, Montreal, Quebec H3S 1Z1 Canada; 2 Centro de Investigación de Enfermedades Tropicales, Universidad Autónoma de Guerrero, Acapulco, Mexico

**Keywords:** Safe birth, Intercultural dialogue, Indigenous health, Fuzzy cognitive mapping

## Abstract

**Background:**

Effective health care requires services that are responsive to local needs and contexts. Achieving this in indigenous settings implies communication between traditional and conventional medicine perspectives. Adequate interaction is especially relevant for maternal health because cultural practices have a notable role during pregnancy, childbirth and the postpartum period. Our work with indigenous communities in the Mexican state of Guerrero used fuzzy cognitive mapping to identify actionable factors for maternal health from the perspective of traditional midwives.

**Methods:**

We worked with twenty-nine indigenous women and men whose communities recognized them as traditional midwives. A group session for each ethnicity explored risks and protective factors for maternal health among the *Me’phaa* and *Nancue ñomndaa* midwives. Participants mapped factors associated with maternal health and weighted the influence of each factor on others. Transitive closure summarized the overall influence of each node with all other factors in the map. Using categories set in discussions with the midwives, the authors condensed the relationships with thematic analysis. The composite map combined categories in the *Me’phaa* and the *Nancue ñomndaa* maps.

**Results:**

Traditional midwives in this setting attend to pregnant women’s physical, mental, and spiritual conditions and the corresponding conditions of their offspring and family. The maps described a complex web of cultural interpretations of disease – “frío” (cold or coldness of the womb), “espanto” (fright), and “coraje” (anger) – abandonment of traditional practices of self-care, women’s mental health, and gender violence as influential risk factors. Protective factors included increased male involvement in maternal health (having a caring, working, and loving husband), receiving support from traditional healers, following protective rituals, and better nutrition.

**Conclusions:**

The maps offer a visual language to present and to discuss indigenous knowledge and to incorporate participant voices into research and decision making. Factors with higher perceived influence in the eyes of the indigenous groups could be a starting point for additional research. Contrasting these maps with other stakeholder views can inform theories of change and support co-design of culturally appropriate interventions.

## Background

Childbirth involves a range of cultural practices and meanings [1] that contribute to women’s perinatal experience and their health outcomes [2]. Many indigenous communities in Latin America have poor access to conventional health services and face harsh living conditions [3]. As we try to understand the dramatic health disparities between indigenous and non-indigenous communities [4], it is difficult to disentangle the effects of poor access to conventional health services from effects of communities losing their own cultures and traditions. There is a need for methods that assess how culture and traditions can impact health outcomes [5].

Effective perinatal care requires services that are responsive to local needs and contexts [6]. Since the 1980s, the concept of cultural safety has gained recognition as a key ingredient in the delivery of quality care, particularly among indigenous communities. Culturally safe practice recognizes that power imbalances shape intercultural interactions and have historical effects on health disparities by influencing the lives and opportunities of marginalized groups [7]. The central idea of cultural safety is to provide health care without diminishing or disrespecting the cultural identity of patients and their communities.

Indigenous communities in Mexico’s Guerrero state lost much of their ancestral traditions as they embraced new elements from Western culture. In transitions like this, in theory people have access to both conventional and traditional health care. In practice, they face complex health choices [8] as the transition from traditional to conventional health care is incomplete in many places, leaving important gaps [9]. Because they usually live in remote parts, many indigenous communities have access only to the very periphery of conventional health services. Distance, inappropriate allocation of state resources, and weak local governments are part of the problem on the supply side [10]. The perceived lack of respect for their traditional knowledge systems leads to an aversion to conventional health services among many indigenous people [11]. This hinders access to conventional medical facilities [12]. In the indigenous communities in the southern mountainous areas of Guerrero in Mexico, traditional midwives are either the only source of perinatal care or the one that women prefer [12, 13].

Traditional midwives are the cornerstone of health care developed over generations by indigenous communities [14]. These systems are culturally specific and have strong links with the environmental conditions grounding each group [15]. Anthropologists have described some elements of traditional health care, mostly using ethnography and interviews [1]. Almost invariably, however, the scientific literature describes these systems from the perspectives of outsiders and using cultural reference points that do not necessarily coincide with those of the indigenous community themselves [16].

Our objective was to systematize the knowledge of traditional midwives about risks and protective factors for maternal health among indigenous communities in southern Mexico, to improve the interface between traditional practitioners and the local health services [17]. The work in this manuscript is part of a bigger project to promote safe birth in cultural safety among indigenous communities in the south of Guerrero State. The overall project includes a cluster randomized controlled trial comparing maternal health outcomes in indigenous communities with and without a co-designed intervention to support the role of traditional midwives [17]. The intervention asserts the principles of cultural safety [18] and intercultural dialogue [19]. The mapping process described in this manuscript will contribute to elicit prior stakeholder knowledge to inform Bayesian analysis of the trial.

## Methods

In recent years, fuzzy cognitive mapping [20] has allowed inclusion of the knowledge of stakeholders into models to describe their understanding of determinants of poor health [21] and, in an additional step, juxtapose this knowledge with conventional biomedicine evidence [22]. These maps describe different knowledge systems and can thus contribute to establishing common reference points to advance shared views of specific health issues [23]. “Fuzzy” refers to the stakeholder assigned weights to grade influences of different factors on each other and on a specific outcome [24]. The maps represent soft models of the way people reason, depicting their knowledge structures [20].

In fuzzy cognitive mapping, each factor is drawn as a node, and each relationship is represented as an edge (arrow) linking nodes. The arrows represent assumptions about causal relationships that can be based on data or on unwritten knowledge [20]. Authors of the maps attribute different values to weight the strength of each arrow. Weights can have positive signs to indicate that, as one node increases, the linked node also increases (excitatory relationship), or negative signs for inhibitory relationships (as one node increases, the linked node decreases). The causal weights express knowledge-holder opinions, their explanatory models and theory of change, rather than a predictive statistical model. By contrasting different stakeholder groups, fuzzy cognitive maps can highlight similarities and differences of alternative explanatory models and theories of change [25].

### Participants

The *Nancue ñomndaa* and *Me’phaa* people have experienced cultural loss associated with the growing Western influence in their area. Nonetheless, both indigenous groups still maintain their identities. This is reflected in the use of traditional languages and, especially in the case of the *Nancue ñomndaa*, clothing. The main economic activities of both indigenous groups are subsistence agriculture, raising cattle, and migrant labor. During the last two decades, these communities have experienced out-migration mainly of male adults and youth looking for jobs in other states, Canada and the United States, to send money back to their families in Guerrero. The minimum wage in the region is about USD40 monthly, but for indigenous populations is around USD34 [13].

Traditional midwives accompany indigenous women throughout pregnancy, provide support through labour and advise on care of the newborn [1, 26, 27]. We recruited 29 indigenous traditional midwives, 18 from the *Me’phaa* indigenous group (Tlapaneco) in the municipality of Acatepec and 11 from the *Nancue ñomndaa* (Amuzgo) indigenous group in the municipality of Xochistlahuaca. A household survey in 2015 interviewed each indigenous woman who had delivered their children in the last two years [17]. The answers allowed us to identify active traditional midwives with *de facto* recognition in their communities, based on the number of births they attended, the health outcomes of their patients, and the traditional knowledge they hold. The traditional midwives invited to the mapping sessions also took part in the intervention of the cluster randomized controlled trial. We invited each midwife in person, as expected in indigenous customs, some weeks before the meeting. All accepted the invitation. The group in Acatepec included two male traditional midwives.

### Drawing the maps

Two community members fluent in both Spanish and the indigenous language who were trained as intercultural brokers [17], two field coordinators from the *Centro de Investigación de Enfermedades Tropicales* (CIET) at the *Universidad Autónoma de Guerrero*, and the lead author facilitated the mapping sessions. After the participants gave their oral informed consent to participate, the lead author gave a further detailed explanation of the mapping steps, using lay language. Participants constructed their maps in one three-hour group session in each indigenous community. The intercultural brokers translated into Spanish the ideas voiced by the traditional midwives. Two additional local translators identified any distortion of the meaning introduced in translation.

Once participants confirmed they understood the mapping process, we invited them to map their answers to the question: To your knowledge, what are the factors related to maternal health in your communities? Each group completed two maps: one of factors that promote safe motherhood (protective factors) and another for factors that impede safe motherhood (risks). Through group discussion, participants first listed the factors they considered to be related to maternal health in their communities. The facilitator wrote each factor on a card and stuck the cards on a wall. Some factors described concepts defined by the participants’ traditional culture. In these cases, the facilitator asked for additional information to clarify the meaning. When no additional factors were forthcoming, the facilitator then asked the participants to identify the causal relationships between factors. The facilitator drew the arrows linking factors and confirmed at each time with the participants that the arrow represented the causal relation they wanted to convey, asking for more details as necessary to understand why they identified that relationship.

After defining all the relationships, participants then ranked the strength of each relationship, using a scale from one to five (with five being the strongest influence, one being the weakest influence). The facilitator explained that the strongest influence (5) was a relationship where the factor in question would almost always cause the linked outcome, while the weakest influence (1) was a relationship where the factor would seldom cause the linked outcome. The midwives decided the weight of each link by consensus. When one irreconcilable difference of opinion about the influence of hospitals occurred, we incorporated this in a sensitivity analysis. An experienced researcher fluent in indigenous language took notes of the explanations and discussion during the session, without recording any personal identifying data about participants. At the end of the session, facilitators took pictures to record the final maps. We used multiple translators to increase the likelihood of capturing the meaning correctly.

### Analysis of the maps

We digitized the maps using the free software yEd [28] and generated a list of nodes and adjacency matrices for the numerical analysis of the relationships. An adjacency matrix presents the structure of the map as a square table with *n* number of rows and *n* number of columns, where *n* equals the total number of nodes. The value of each cell is the weight of the relationship between two nodes (directed from the row to the column). For the matrices of the original maps, we scaled the weights 1 to 5 by dividing all with a constant 5.

For each original map, we calculated the fuzzy transitive closure [29] between nodes, to measure the influence each node had on others in the map. Transitive closure takes account of each pair of linked concepts in the context of all the possible connections in the map. A “walk” is any succession of edges (arrows) that allows transit from one node to another. The value of the fuzzy transitive closure between two nodes A and B is the maximum weight of any of the walks from A to B, and the weight of each walk is the minimum weight of any of the edges (arrows) involved in the walk. After transitive closure, the maps had a new architecture that included all the possible connections between nodes, with values from 0 to 1 representing the strength of the influence (with one being the highest influence) and positive or negative signs to represent excitatory and inhibitory relationships respectively. After transitive closure, we combined the maps using a weighted average of the strength of the influences [23]. The weight assigned to each map was the cumulative experience of the midwives who made it, defined by the number of them in each.

We used thematic analysis to condense the concepts (nodes) into fewer categories to facilitate the communication of the content [30, 31]. The lead author developed a first level of aggregation using a pattern matching table to arrange the nodes of each map with similar meanings and their corresponding categories (Table 1). Each factor represented an idea that was discussed and agreed upon, with traditional midwives clarifying the words and specifying their meaning. Identifying categories from factors across maps thus incorporated those deeper meanings described in the notes from the mapping session. A group of researchers with extensive experience with indigenous communities in Guerrero, including two who participated in the mapping sessions, confirmed the categories developed in the first aggregation (SP, NA, AC, Abraham de Jesús García, Nadia Maciel Paulino, and Germán Zuluaga). In a member checking exercise [32] in July 2018, IS presented the maps to the traditional midwives who confirmed their agreement with the results of the analysis.

**Table 1:** Matching table of the concepts grouping the risk and protective factors

**Risk factors in Acatepec**	**Risk factors in Xochistlahuaca**
*Category: The woman does not have a healthy maternity (nor a healthy delivery)*	
The woman suffers “Espanto” (fright)	The woman suffers “Espanto” (fright) (traditional disease)
The woman suffers “Antojo”/Craving	The woman suffers “Antojo”/Craving (traditional disease)
The woman suffers “Shaime” (traditional disease)	
The woman suffers “Smoke” (traditional disease, different from smoking)	
The woman suffers “The evil eye” (traditional disease)	
	The woman suffers “Nahual” (traditional disease)
	The woman suffers “Coraje” (anger) (traditional disease)
	The baby suffers “Nquio” (traditional disease)
Woman’s body and face swelling	Woman’s feet swelling, abdominal swelling
Cold / Coldness of the womb	Cold / Coldness
Hemorrhage (pregnancy)	Bleeding (pregnancy)
Headache (pregnancy)	
Decreased appetite	
Chills (fever and cold)	
Cough	
	Flatulence
	Seizures
	Weight loss
	Vaginal discharge, itching
	Dizziness, nausea, vomiting (during pregnancy and delivery)
	Painful labor and delivery
	Vaginal swelling (delivery)
Breech presentation (delivery)	
Baby wrapped in umbilical cord (delivery)	
Prolonged labor	Prolonged labor
Tiredness (delivery)	Fatigue (delivery)
	Seeing flashing lights (delivery)
	Faint during delivery
Headache	Headache (delivery)
Hemorrhage during delivery	Hemorrhage during delivery
Retained placenta	Retained placenta
*Category: The woman dies*	
Maternal Death	Maternal Death
*Category: The baby dies*	
Infant death	Infant death
Pregnancy loss	
*Category: Abnormal position of baby*	
Abnormal position of baby	Abnormal position of baby
*Category: Abortion*	
Abortion	Abortion
*Category: The woman suffers violence*	
Violence (partner or family, sexual abuse, absent father, extramarital children, treats from the father to make her abort)	Violence (domestic violence related with alcohol consumption)
	Disagreement or fight
*Category: Unsupportive family environment*	
Unsupportive family environment	
*Category: The woman does not follow protective rituals*	
Not following protective rituals (lighting candles in the mountain or prayers)	
*Category: The woman does not follow self-care practices*	
Practices such as: cooking too close to the fire, using long thread when sewing.	Practices such as: carrying heavy loads, shower with cold water, eating cold tortillas, eating pork, eating too much chili pepper, or not covering the head after delivery.
Eating forbidden food (a long list of fruits and animals)	
The woman has multiple sexual partners	
Shower with cold water	
Expose to cold environments	
Heavy work	
	Poor hygiene
	Ignorance of when to push
	Wrong position while sleeping
	Sexual relations too early after delivery
	Drinking alcohol (getting drunk) and infidelity
*Category: Accidents*	
Accidents	
Poisonous animal bites	
*Category: Intended spiritual attacks from others*	
Intended spiritual attacks from others	
Envy	
*Category: Physical or spiritual imbalance*	
Someone with “heavy” sight looks the women	
	Physical or spiritual imbalances
*Category: Primigravida*	
Primigravida	
*Category: The woman has poor health condition (before pregnancy)*	
	The woman has “weak blood”
*Category: The woman is poorly nourished*	
Bad nutrition	Bad nutrition
*Category: The woman has worries, feels disgust or nervous during pregnancy*	
The woman feels nervous during pregnancy	
The woman has fright caused by thunders, animals, or accidents	The woman has fright
The woman feels embarrassment or sadness	
	The woman finds something disgusting
*Category: Unwanted pregnancy*	
Unwanted pregnancy	Unwanted pregnancy
**Protective factors enumerated in Acatepec**	**Protective factors enumerated in Xochistlahuaca**
*Category: The woman has a safe birth and healthy maternity*	
The woman is happy	The woman is happy, beautiful, good worker, not lazy, does not get “coraje” (anger). Also, she has a healthy husband
The woman is strong and brave	
The woman is able to give birth at home	A good labor and delivery: healthy pains, less blood loss, fast healing
The woman does not get sick	Healthy postpartum: healthy baby / the woman is willing to eat after labor
*Category: The woman has support of a traditional midwife or healer*	
Support of a midwife or traditional healer	The woman receives care from the traditional midwife (and she takes care of the position of the baby)
	Traditional midwives in the community
A midwife counsels the husband	
*Category: Healthcare center or hospital is available*	
Healthcare centers available	Hospital available (Hospital básico comunitario)
*Category: The woman follows protective rituals*	
The woman follows protective rituals (lighting candles or indigenous prayers)	The woman follows protective rituals associated with traditional medicine
Praying in the church (Cristian or Catholic) asking for health	
*Category: The woman follows self-care practices*	
	The woman takes care of herself
*Category: The woman does not suffer violence*	
The woman does not suffer violence	
*Category: The woman lives without worries*	
	The woman lives without worries
*Category: The woman has a caring, working, and loving husband*	
	The woman is well treated by the husband
The woman has a caring and loving husband	The woman has a caring and working husband
	The husband talks to the baby in the womb
*Category: The woman has good communication with husband*	
	Good communication with husband
	The woman discusses (talks) with husband about pregnancy and delivery
*Category: The woman has a good health condition (before pregnancy)*	
	The woman does not get sick
	The woman heals from her diseases
*Category: The woman has economic stability*	
	Economic stability
*Category: The woman is well nourished*	
The woman eats good (enough) food	The woman eats good (enough) food

Using the aggregation categories, we described similarities and differences of maps from each municipality (Table 2). A formal comparison between maps identified: (a) validated connections (both maps share the non-zero connection with the same sign), (b) non-validated connections (it is only mentioned in one map), and (c) conflicting connections (both maps include the edge but with different directions). We summarized the cumulative net influence of each category from the thematic analysis as a proportion of total weight for each factor in two steps. First, we calculated the cumulative weight for each category as the sum of weights of the influences of the factors in the transitive closure maps in the corresponding category. Second, we divided each cumulative weight by the maximum total cumulative weight across all the categories in the synthesis map. As a measure of the overall agreement in the cumulative net influence, we divided the total size of all differences (summation of the absolute value of the differences) by the number of differences. An average difference closer to one indicates less agreement about the weight of the relationships.

**Table 2:** Pattern marching table of the cumulative net influence of each category on maternal health

Risk factors							Protective factors						
***Me’phaa*** Acatepe		***Nancue ñomndaa*** Xochistlahuaca				Final map	***Me’phaa*** Acatepe		***Nancue ñomndaa*** Xochistlahuaca				Final map
Factors	CNI	Factors	CNI	Validation	Difference	CNI	Factors	CNI	Factors	CNI	Validation	Difference	CNI
*Category: The woman does not have a healthy maternity (nor a healthy delivery)*							*Category: The woman has a safe birth and healthy maternity*						
17	0.29	23	1.00	Val.	0.71	0.76	4	0.00	3	0.30	Val.	0.30	0.18
*Category: The woman dies*													
1	0.00	1	0.00	Val.	0.00	0.00							
*Category: The baby dies*													
2	0.00	1	0.00	Val.	0.00	0.00							
*Category: The woman suffers violence*							*Category: The woman does not suffer violence*						
1	0.11	2	0.46	Val.	0.35	0.34	1	0.50	0	0.00	Nval.	0.50	0.24
*Category: The woman has worries, feels disgust or nervous during pregnancy*							*Category: The woman lives without worries*						
3	0.29	2	0.18	Val.	0.11	0.30	0	0.00	1	0.36	Nval.	0.40	0.22
*Category: The woman does not follow protective rituals*							*Category: The woman follows protective rituals*						
1	0.11	0	0.00	Nval.	0.11	0.07	2	1.00	1	0.36	Val.	0.60	0.70
*Category: The woman does not follow self-care practices*							*Category: The woman follows self-care practices*						
6	1.00	6	0.71	Val.	0.29	1.00	0	0.00	1	0.36	Nval.	0.40	0.22
*Category: The woman has poor health condition (before pregnancy)*							*Category: The woman has a good health condition (before pregnancy)*						
0	0.00	1	0.07	Nval.	0.07	0.04	0	0.00	2	0.73	Nval.	0.70	0.44
*Category: The woman is poorly nourished*							*Category: The woman is well nourished*						
1	0.04	1	0.09	Val.	0.05	0.08	1	0.81	1	0.42	Val.	0.41	0.65
*Category: Abnormal position of baby*													
3	0.11	1	0.02	Val.	0.09	0.08							
*Category: Abortion*													
1	0.04	1	0.00	Val.	0.04	0.02							
*Category: Unsupportive family environment*													
1	0.11	0	0.00	Nval.	0.11	0.07							
*Category: Accidents*													
2	0.04	0	0.00	Nval.	0.04	0.02							
*Category: Intended spiritual attacks from others*													
2	0.21	0	0.00	Nval.	0.21	0.12							
*Category: Physical or spiritual imbalance*													
1	0.04	1	0.21	Val.	0.17	0.15							
*Category: Primigravida*													
1	0.04	0	0.00	Nval.	0.04	0.02							
*Category: Unwanted pregnancy*													
1	0.04	1	0.00	Val.	0.04	0.02							
							*Category: The woman has support of a traditional midwife or healer*						
							2	0.94	2	0.79	Val.	0.14	0.93
							*Category: Healthcare center or hospital is available*						
							1	−0.13	1	0.36	Con.	0.43	0.16
							*Category: The woman has a caring, working, and loving husband*						
							1	0.81	3	1.00	Val.	0.19	1.00
							*Category: The woman has good communication with husband*						
							0	0.00	2	0.73	Nval.	0.70	0.44
							*Category: The woman has economic stability*						
							0	0.00	1	0.33	Nval.	0.30	0.20
**44**		**41**			**0.14**	**–**	**12**		**18**			**0.42**	**–**

# factors: number of factors included in the category; Validation: *Val* validated, *Nval* non-validated, *Con* conflictive; *CNI* cumulative net influence by municipality and final map. Difference: absolute value of the difference between CNI in the two municipalities

## Results

The traditional midwives from Acatepec described unsafe maternity as a set of traditional diseases that can affect women, symptoms associated with those diseases, and events that affect the women and their babies’ health and well-being. They included two additional categories to describe the concrete events of maternal and infant deaths. When describing safe maternity, in addition to not having a disease, they emphasized the happiness and confidence of the women. Traditional views characterized a healthy woman as one who can give birth at home. In a similar integrated approach to healthy maternity, midwives in Xochistlahuaca explicitly included as outcomes in this category the health status of the offspring and even the health status of the husband.

### Risk factors

**Fig. 1:**
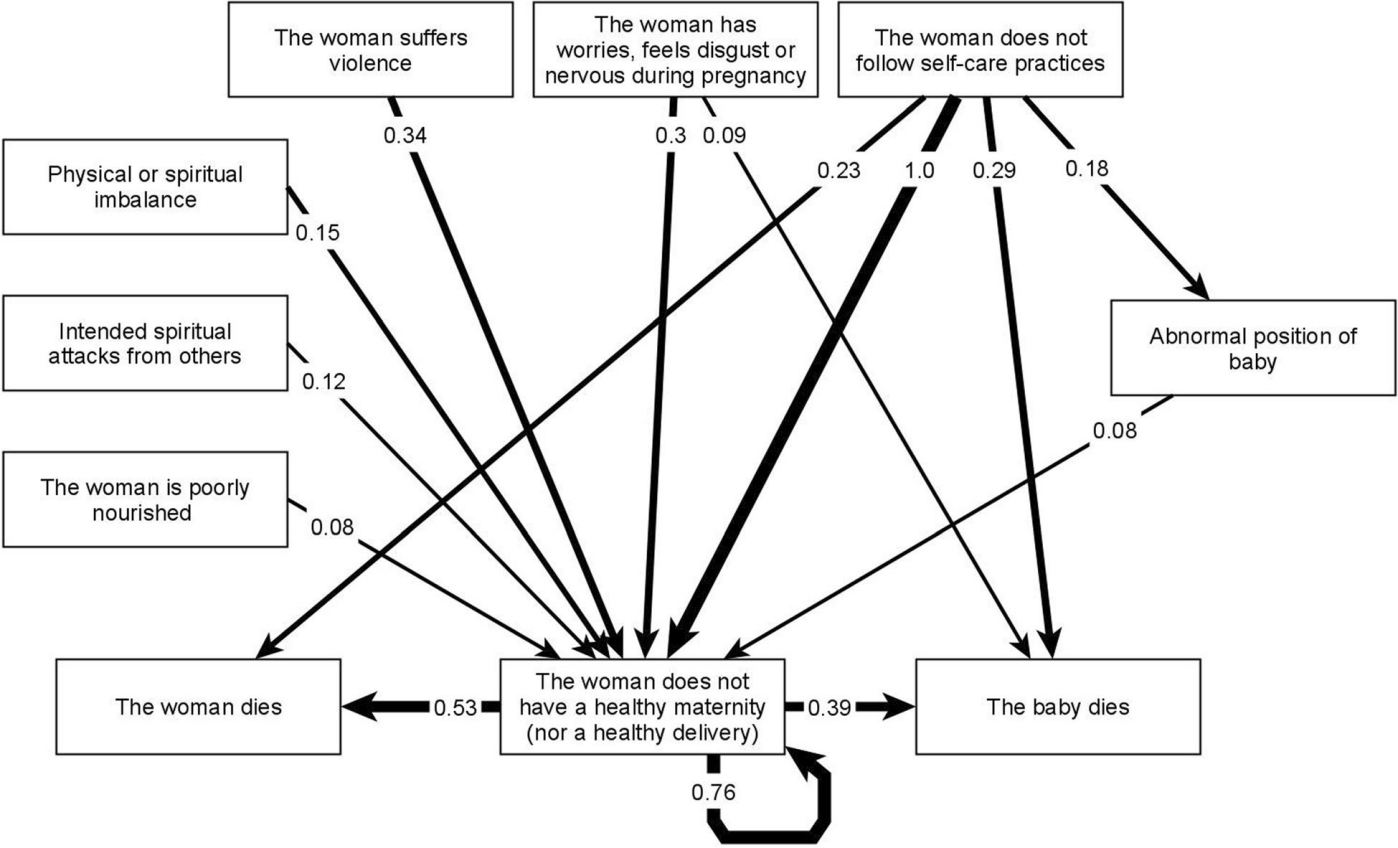
Fuzzy cognitive map of the most influential categories of risk factors. To simplify the graph, we only included the highest-weighted relationships. Additional file 1 contains all the relationships on the map. Strong lines represent excitatory relationships. The numbers on the edges represent the cumulative net influence of one category on another, where 1 is the highest influence in the map

In the map from Acatepec, participants described 44 risk factors (nodes) with 87 relationships (edges). Xochistlahuaca traditional midwives included 42 nodes and 87 edges. The thematic analysis grouped the nodes into 17 categories of risk factor. Table 1 presents the factors included in each category. Factors with the same meaning in both municipalities align in the same row. Figure 1 presents the fuzzy cognitive map of categories with the highest cumulative net influence. The full adjacency matrix with all the relationships for this map is available as Additional file 1.

The most influential category of risk for unsafe maternity was “not following self-care practices” as defined in the customs and traditions of these communities. These practices can include dietary restrictions, reduction of heavy work, less exposure of mother’s body to cold water, or hygiene practices. Midwives from both communities included this category, although the actual contents of these practices are heterogeneous and could be culture specific. During thematic analysis, the researchers recognized that factors in other categories (such as rituals or nourishment) could also correspond to self-care practices, which would increase their relevance within the system. This category appeared as protective in Xochistlahuaca (“The woman follows self-care practices”), but not explicitly mentioned in the Acatepec protection map. Among the risk categories, the midwives identified gender violence and mental health of women (“The woman has worries, feels disgust or nervous during pregnancy”) as highly influential (second and third order importance respectively). They described an unsupportive family environment as a cause of violence against women.

In the final map, the multi-concept category “the woman does not have a healthy maternity” has a self-pointing edge with a cumulative net influence of 0.76 (Fig. 1). This loop, from the node back to itself, implies that factors within the category influence other factors grouped in the same category. We reviewed the initial maps to identify concepts with greater influence within the category. Three factors showed a strong influence in maternal health outcomes, “cold or coldness of the womb”, “espanto” (literally translated as fright), and “coraje” (literally translated as anger). They also had a strong influence on maternal and infant death. Both indigenous groups confirmed “coldness of the womb” and “espanto”, but “coraje” was a specific factor for the *Nancue ñomndaa* from Xochistlahuaca (Table 2). Even with translation, the words do not hold an equivalent meaning in English or Spanish. Traditional midwives explained that “coldness of the womb” resulted from exposing the mother’s body to cold elements such as water, fresh air, or certain foods considered of cold nature. They explained the womb needs to remain warm to allow for the correct development of the baby and to function properly during delivery. The concept of “espanto” (fright) describes a strong emotional impact that alters one’s mental health. Examples include violence, an animal attack, or an accident. They explained that “coraje” (anger) as caused by an imbalance produced by violence, not necessarily directed at the woman, that affects the “aire” (air) or environment of the mother and consequently affects her health.

### Protective factors

**Fig. 2:**
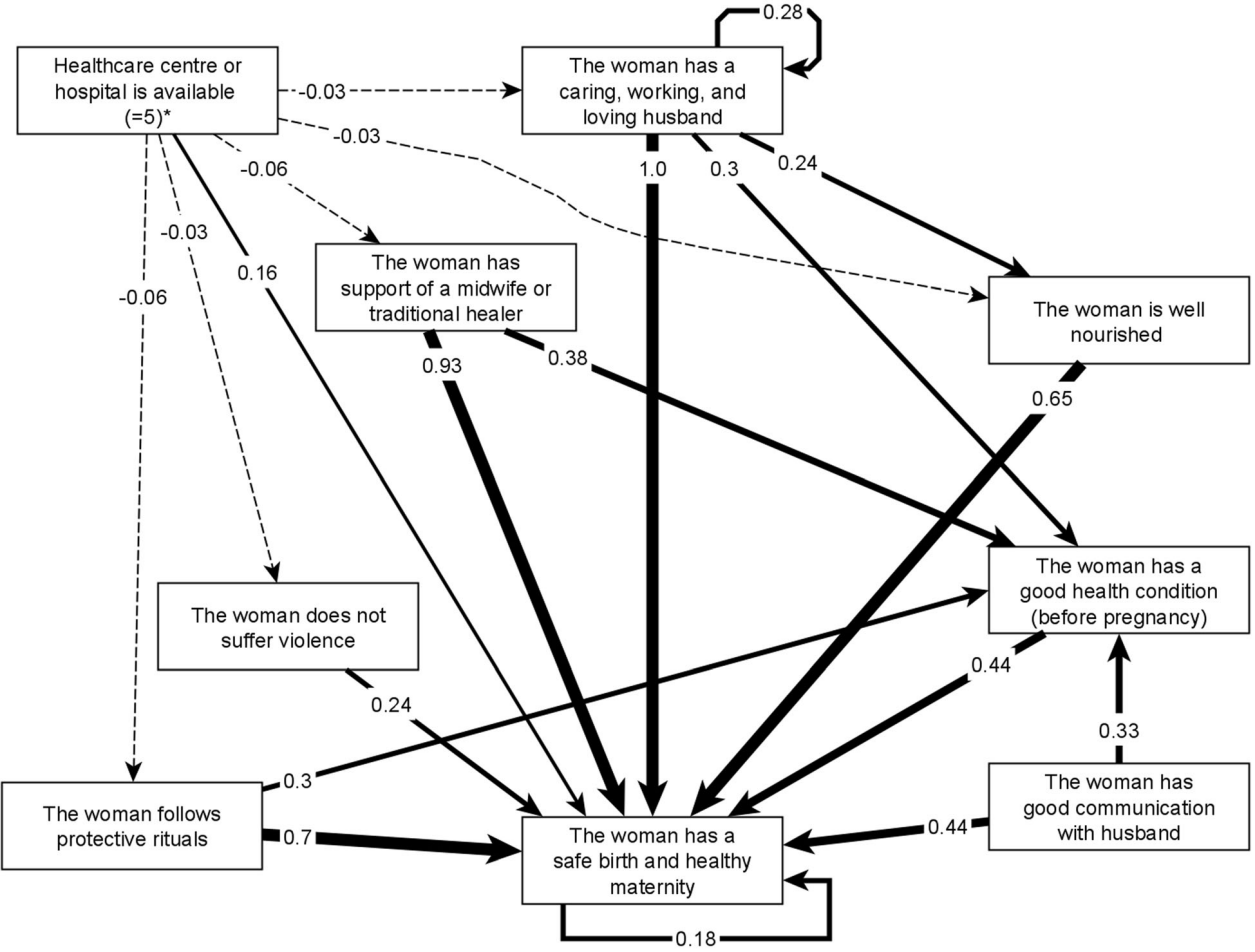
Fuzzy cognitive map of the most influential categories of protective factors on maternal health. To simplify the graph, we only included the highest-weighted relationships. Additional file 2 contains all the relationships on the map. Strong lines represent excitatory relationships and dashed lines represent inhibitory relationships. The numbers on the edges represent the cumulative net influence of one category on another, where 1 is the highest influence in the map. For this map we used the maximum positive influence reported by participants for the role of hospitals and health centers

In Acatepec, traditional midwives reported 12 protective factors (nodes) with 38 relationships while in Xochistlahuaca, traditional midwives included in their map 18 nodes and 31 relationships. The thematic analysis condensed the protective factors into 12 shared categories (Table 1). Figure 2 presents the map of the strongest protective factors and Additional file 2 has the full adjacency matrix with all the relationships among categories. Protection maps highlighted the importance of male support (described as having a caring, working, and loving husband) and support from traditional midwives in promoting maternal health. Midwives in both municipalities mentioned both these two factors (Table 2). They rated protective rituals and access to adequate food for pregnant women in third and fourth place for influence. The map also showed the influence of protective factors over the intermediate outcome of women’s health condition before pregnancy (Fig. 2, category P10 in Additional file 2).

In line with the risk map, the map of protective factors showed non-exposure to violence as a strong influence. The map showed how other factors were protective through decreasing the levels of violence that women experience. These factors included counseling by traditional midwives, protective rituals, access to food, economic stability, and having a caring husband. Having a caring husband was validated across both indigenous groups. The map of protectors included other “mirror images” of risk categories for mental health of women, practicing protective rituals and self-care practices, good nutrition and health condition of the women before pregnancy (at the top of Table 2).

One category, “Healthcare center or hospital is available”, had a conflictive validation. Acatepec midwives showed it as a negative influence on safe maternity whereas it was a positive influence in Xochistlahuaca, where it was the only relationship for which participants did not reach consensus (Additional file 2). Individual traditional midwives weighted its protective influence on women’s health between no protective effect at all (0) and a high positive effect (5). Per protocol, we sought reasons for this divergence: one participant wanted to assign a 5 and the others were discussing between 0 and 1. The participant who suggested a weight of 5 was a very experienced traditional midwife who was well-respected by the medical staff at the healthcare center, suggesting that strong inter-professional and cross-cultural relationships can greatly change the role that healthcare centers can play in indigenous communities. Additional file 2 includes an additional row to present the variation of the cumulative influence when assuming a positive effect of five or no-effect in the map from Xochistlahuaca. The negative effect assigned in the map from Acatepec not only affected safe maternity, but also had negative impacts on other categories, particularly those related with the services of traditional practitioners, following traditional rituals, male involvement, violence against women, and access to food (dashed lines in Fig. 2). These effects did not emerge in the Xochistlahuaca map.

## Discussion

We used fuzzy cognitive mapping to document traditional indigenous knowledge related to maternal health. FCM is particularly useful in multicultural contexts, as it can be used across language barriers and educational levels [20]. Fuzzy cognitive mapping offered a transparent and systematic way to organize and to summarize indigenous views despite intercultural differences. Traditional midwives described a broad understanding of maternal health that included their well-being and their surroundings. This comprehensive approach to health highlights the need for better indicators, measures, and benchmarks to assess quality of care [33]. We will use the models to support discussion of future actions to promote maternal health with health providers and community members.

The views of indigenous traditional midwives on maternal health in their communities included a complex set of concepts and relationships. Prominent among the risk factors mentioned by the traditional midwives were failure to follow traditional practices of self-care, those associated with cultural concepts of disease (“espanto” (fright), “coraje” (anger), and “coldness of the womb”), and women’s mental health and experience of violence. Among the protective factors, male involvement (having a caring, working, and loving husband), support of traditional healers, protective rituals and adequate nourishment were most influential.

The literature is replete with examples of traditional practices for childbirth and maternal health [34–39]. Traditional practices associated with maternal health are best viewed as complex interventions with many interacting aspects. This makes it difficult to tease out the key element in any change [40]. Despite this lack of understanding, potential benefits or harms of these practices are usually defined authoritatively from a conventional medicine perspective [41]. A cultural gap prevents many of us going beyond initial judgements of implausibility based on Western worldviews. This in turn hampers research on the etiology, symptoms, and indigenous health concerns [1]. Methods like FCM can help to document and interpret traditional practices, thus helping to bridge this gap [16, 42]. With these methods in hand, Western epistemological frameworks need not go unchallenged in intercultural settings [43, 44].

The culturally specific conditions listed by the traditional midwives are not limited to pregnancy and childbirth. A study of Mexican populations in the United States associates “espanto” (fright in English also called *susto* in Spanish) with the onset of type 2 diabetes [45]. Other studies present “espanto” as the somatic expression of psychiatric disorders, often as a consequence of domestic violence or other traumatic experiences [46]. And some other authors see these diseases as physical consequences of unfulfilled social expectation, inequities, or harsh environmental conditions [47–49]. The cold-hot dichotomy associated with “coldness of the womb” is a theory of disease etiology found in traditional health systems of indigenous groups in the Americas, Africa, Europe and Asia [40]. The concept is complicated by the relative independence from temperature as understood in conventional medicine [50]. Recent reports suggest an association, however, between this indigenous classification of diseases and physical responses to chemical stimuli of medicinal plants for their treatment [51].

Traditional midwives promote male involvement and increase family and community support for women. Supporting them in this role can use existing cultural dynamics to promote positive change, for example to decrease domestic violence [52]. Reducing the role of traditional midwives to “birth attendants” ignores the crucial fact that they also work as counselors of women, men, families and communities in general. Even those who advocate replacing traditional midwives with practitioners trained in conventional medicine acknowledge it is worth keeping positive aspects of their role: “the sense of caring, the human approach, and the response to cultural and spiritual needs” [53].

The map of protective factors also highlighted traditional rituals of fertility and proper nourishment of women. The health effects of traditional rituals remains an unexplored field with significant methodological challenges, mainly associated with the multifactorial nature of these interventions [5, 54], as we have explained before for the category of self-care practices. Poor nutrition is an important concern for populations like those in our study, who have a disproportionately lower income, depend on subsistence agriculture, and have been displaced to less productive land. Poor nutritional indicators are common among indigenous communities [55], which often suffer from structural inequities [56]. Cultural continuity and preservation of local resources, both goals of a culturally safe approach, can improve food security among indigenous groups [57].

### Strengths and limitations

The advantages of FCM are several. It takes only a short time necessary to summarize a lot of information. The graph language facilitates data collection, analysis, and interpretation across cultural, language and educational barriers, and it is easily adjusted for different knowledge systems [20]. It can take into account complex socio-cultural mechanisms that effect the well-being of women, offspring and communities [33]. It is easy to share knowledge in an accessible form to facilitate discussion with others and can facilitate intercultural dialogue [19] to improve the interface of indigenous communities with conventional medicine.

In research, fuzzy cognitive mapping helps to summarize participant views of causality. The maps can identify theories of change and frame hypotheses for empirical research and decision making. The bigger project with indigenous communities in southern Guerrero used a parallel group randomized controlled trial to test some of the causal relationships in the maps, particularly the influence of traditional midwifery on health outcomes [17]. The maps also opened opportunities for evidence-based conversations to deepen our understanding of the factors involved in safe birth [58].

One risk category defined with the midwives to consolidate the maps turned out to be larger than other categories and it included what seemed like heterogeneous factors. At first glance, for example, “coldness of the womb” seems very different from “hemorrhage”. But for traditional midwives hemorrhage is the outcome of coldness and it can lead to the death of a woman. Category maps are models of individual concepts generalized to a larger scale, which simplify the contents to facilitate communication. But scale matters, and interpretation of maps has to follow the level of generalization of the model [59]. We cannot assume that relationships between categories apply equally to all the factors within those categories. Doing so would constitute a cross-level fallacy [59, 60]. It is possible to unpack aggregated category maps by going back to the transitive closure maps to identify specific paths through which individual factors influence each other.

Interpretation across languages is a challenge in most intercultural settings, especially when full translation is not practical (as in a group discussion). As researchers, we made several assumptions during the thematic classification of factors and the overall weight assigned to the maps from the two groups to calculate the weighted average. We documented these assumptions so their impact in the analysis can be assessed. Member checking with the authors of the maps encouraged us to believe that researcher assumptions during the analysis did not contradict the meaning of the information the traditional midwives provided. The mapping exercise took place in the context of years of work and trust building with the communities concerned, and it was greatly helped by the involvement of local personnel with skills and experience in intercultural dialogue. Implementing a similar exercise in settings without a history of collaboration would be challenging.

## Conclusions

Fuzzy cognitive mapping provided a robust way to summarize and to value the complex knowledge of indigenous midwives. In our example, the maps identified locally relevant cultural concepts related to maternal health in Guerrero State. Better understanding of these could promote collaboration and help to defuse disagreements between conventional health services and indigenous communities; thus, increasing the effectiveness of perinatal care in those disadvantaged communities.

More broadly, fuzzy cognitive mapping is a tool for indigenous and other marginalized communities to communicate their way of seeing things to health authorities and to open discussions about health initiatives. In combination with maps from other sources, such as researchers or published literature, the maps can be used to develop composite theories of change. They can identify key factors for inclusion in questionnaires and to frame health outcomes and weight stakeholder prior beliefs to serve in Bayesian analysis. From clarifying the causal concepts through to formal statistical analysis, fuzzy cognitive mapping helps to build the voices of indigenous participants into modern health research.

## Supplementary information

**Additional file 1.** Adjacency matrix of the final map showing categories of risk factors for maternal health in the South of Guerrero.

**Additional file 2.** Adjacency matrix of the final map showing categories of protective factors for maternal health in the South of Guerrero.

## Data Availability

The datasets generated during or analyzed during the current study will be available upon request from CIET. Before the information can be shared, the requester will need to present a plan for data analysis. Also, the requester will need to complete the procedure for ethical approval of the secondary analysis in accordance with the procedures defined by the Ethics Board of the *Universidad Autónoma de Guerrero* and the agreements with communities to ensure the protection of the participants.

## Abbreviations

CIET: *Centro de Investigación de Enfermedades Tropicales*
FCM: Fuzzy cognitive mapping
