# Supplementary material for: Fuzzy cognitive mapping and soft models of indigenous knowledge on maternal health in Guerrero, Mexico
Source: BMC Med Res Methodol. 2020 May 19;20:125. doi: 10.1186/s12874-020-00998-w (PMC7238543; doi:10.1186/s12874-020-00998-w)
Supplement: Supplementary file 1 — Additional file 1. Adjacency matrix of the final map showing categories of risk factors for maternal health in the South of Guerrero. [file 12874_2020_998_MOESM1_ESM.docx]

Additional File 1

Adjacency matrix for the map of risk categories after combination of *Me’phaa* and *Nancue ñomndaa* perspectives

|  | R1 | R1a | R1b | R2 | R3 | R4 | R5 | R6 | R7 | R8 | R9 | R10 | R11 | R12 | R13 | R14 | R15 |
| --- | --- | --- | --- | --- | --- | --- | --- | --- | --- | --- | --- | --- | --- | --- | --- | --- | --- |
| R1 The woman does not have a healthy maternity (nor a healthy delivery) | 0.76 | 0.53 | 0.39 | 0.01 |  |  |  |  |  |  |  |  |  |  |  |  |  |
| R1a The woman dies |  |  | 0.01 |  |  |  |  |  |  |  |  |  |  |  |  |  |  |
| R1b The baby dies |  |  |  |  |  |  |  |  |  |  |  |  |  |  |  |  |  |
| R2 Abnormal position of baby | 0.08 | 0.03 | 0.01 |  |  |  |  |  |  |  |  |  |  |  |  |  |  |
| R3 Abortion | 0.02 | 0.06 | 0.03 |  |  |  |  |  |  |  |  |  |  |  |  |  |  |
| R4 The woman suffers violence | 0.34 | 0.07 | 0.07 |  | 0.03 |  |  |  |  |  |  |  |  |  |  |  |  |
| R5 Unsupportive family environment | 0.07 | 0.02 | 0.05 |  | 0.02 | 0.02 |  |  |  |  | 0.02 |  |  |  |  |  |  |
| R6 The woman does not follow protective rituals | 0.07 | 0.02 | 0.02 |  |  |  |  |  |  |  |  |  |  |  |  |  |  |
| R7 The woman does not follow self-care practices | 1.00 | 0.23 | 0.29 | 0.18 | 0.01 | 0.01 |  |  |  |  |  |  |  |  |  |  |  |
| R8 Accidents | 0.02 | 0.05 | 0.05 |  |  |  |  |  |  |  |  |  |  |  |  |  |  |
| R9 Intended spiritual attacks from others | 0.12 | 0.05 |  | 0.05 |  |  |  |  |  |  | 0.02 |  |  |  |  |  |  |
| R10 Physical or spiritual imbalance | 0.15 | 0.03 | 0.01 |  |  |  |  |  |  |  |  |  |  |  |  |  |  |
| R11 Primigravida | 0.02 | 0.02 | 0.02 |  |  |  |  |  |  |  |  |  |  |  |  |  |  |
| R12 The woman has poor health condition (before pregnancy) | 0.04 | 0.01 | 0.01 |  |  |  |  |  |  |  |  |  |  |  |  |  |  |
| R13 The woman is poorly nourished | 0.08 | 0.01 | 0.01 |  |  |  |  |  |  |  |  |  |  | 0.01 |  |  |  |
| R14 The woman has worries, feels disgust or nervous during pregnancy | 0.30 | 0.05 | 0.09 |  |  |  |  |  |  |  |  |  |  |  |  |  |  |
| R15 Unwanted pregnancy | 0.02 | 0.03 | 0.03 |  | 0.03 |  |  |  |  |  |  |  |  |  |  |  |  |

The numbers in the cells represent the cumulative net influence of one category on another, where 1 is the highest influence in the map. Positive and negative signs represent excitatory and inhibitory relationships respectively.
