# Supplementary material for: Fuzzy cognitive mapping and soft models of indigenous knowledge on maternal health in Guerrero, Mexico
Source: BMC Med Res Methodol. 2020 May 19;20:125. doi: 10.1186/s12874-020-00998-w (PMC7238543; doi:10.1186/s12874-020-00998-w)
Supplement: Supplementary file 2 — Additional file 2. Adjacency matrix of the final map showing categories of protective factors for maternal health in the South of Guerrero. [file 12874_2020_998_MOESM2_ESM.docx]

Additional File 2

Adjacency matrix for the map of protective categories after combination of *Me’phaa* and *Nancue ñomndaa* perspectives

|  | P1 | P2 | P3 | P4 | P5 | P6 | P7 | P8 | P9 | P10 | P11 | P12 |
| --- | --- | --- | --- | --- | --- | --- | --- | --- | --- | --- | --- | --- |
| P1 The woman has a safe birth and healthy maternity | 0.18 |  |  |  |  |  |  |  |  |  |  |  |
| P2 The woman has support of a midwife or traditional healer | 0.93 | 0.15 |  | 0.03 |  | 0.15 |  | 0.15 | 0.15 | 0.38 |  | 0.15 |
| P3 Healthcare center or hospital is available (= 5)* | 0.16 | -0.06 |  | -0.06 |  | -0.03 |  | -0.03 |  | 0.12 |  | -0.03 |
| P3 Healthcare center or hospital is available (= 0)* | 0.11 | -0,06 |  | -0,06 |  | -0,03 |  | -0,03 |  | 0,03 |  | -0,03 |
| P4 The woman follows protective rituals | 0.70 |  |  |  |  | 0.15 |  | 0.06 |  | 0.30 |  | 0.06 |
| P5 The woman follows self-care practices | 0.22 |  |  |  |  |  |  |  |  | 0.09 |  |  |
| P6 The woman does not suffer violence | 0.24 |  |  |  |  |  |  |  |  | 0.12 |  |  |
| P7 The woman lives without worries | 0.22 |  |  |  |  |  |  |  |  |  |  |  |
| P8 The woman has a caring, working, and loving husband | 1.00 |  |  |  |  | 0.09 | 0.18 | 0.28 |  | 0.30 | 0.04 | 0.24 |
| P9 The woman has good communication with husband | 0.44 |  |  |  |  |  |  |  | 0.09 | 0.33 |  |  |
| P10 The woman has a good health condition (before pregnancy) | 0.44 |  |  |  |  |  |  |  |  |  |  |  |
| P11 The woman has economic stability | 0.20 |  |  |  |  |  | 0.07 |  |  |  |  | 0.06 |
| P12 The woman is well nourished | 0.65 |  |  |  |  | 0.09 |  |  |  | 0.15 |  |  |

The numbers in the cells represent the cumulative net influence of one category on another, where 1 is the highest influence in the map. Positive and negative signs represent excitatory and inhibitory relationships respectively.

* Calculated for the two extreme values discussed in Xochistlahuaca.
